# Supplementary material for: Functional aspects of the Eustachian tube by means of 3D-modeling
Source: PLoS One. 2021 Aug 12;16(8):e0244909. doi: 10.1371/journal.pone.0244909 (PMC8360510; doi:10.1371/journal.pone.0244909)
Supplement: Attachment — Submitted filename: Response to Academic Reviewers.docx [file pone.0244909.s001.docx]

Dear Academic Reviewers,

Thank you very much for the sound and valuable suggestions, which led to a clarification and extension of the paper.

Best regards

Reviewer #1: Thank you very much for the opportunity to review the manuscript titled "Functional Aspects of the Eustachian Tube by Means of 3D-Modeling". I congratulate the authors for this quite interesting study.

Reviewer #2: The presen study makes a contribution to the current litereature providing an analysis on the structure of ET using both histopathological and radiological 3D- reconstruction models. Cons of the study is that only one sample was investigated, and it was not a human sample. Pros of the study are the effort on the methodology, and information provided based on the structure of the ET. In the big picture, it deserves publication. However, in the prevoous literature, there exist a few studies (from the laboratory of Michael Paparella) conducted on human temporal bones, constituting 3d reconstruction models. I thimk these publications deserve citation:
This has been implemented: Cit. 11, 25 an 29

1- Epitympanum volume and tympanic isthmus area in temporal bones with retraction pockets.
Monsanto RD, Pauna HF, Kaya S, Hızlı Ö, Kwon G, Paparella MM, Cureoglu S.
Laryngoscope. 2016 Nov;126(11):E369-E374. doi: 10.1002/lary.25937. Epub 2016 Apr 23.
PMID: 27107158

Cited paper

2- A three-dimensional analysis of the endolymph drainage system in Meniere disease.
Monsanto RD, Pauna HF, Kwon G, Schachern PA, Tsuprun V, Paparella MM, Cureoglu S.
Laryngoscope. 2017 May;127(5):E170-E175. doi: 10.1002/lary.26155. Epub 2016 Jul 21.
PMID: 27440440 Free PMC article.

Cited paper

Reviewer #3: This reviewer congratulates the authors in this meticulous work and great presentation. The points below will enhance the importance of this manuscript.
This reviewer is looking forward to their similar work on human cadaver specimens.
Line 87- TVPM is not parallel to the bony part of the ET. It is almost parallel with the cartilaginous part.

the tensor tympani muscle was meant, not the tensor veli palatini muscle

Line 202. Authors refer to a hypothesis here, but it is not clear what this refers to. They discuss the current literature on functional aspects and implications of these anatomical relationships. But they have not clearly identified any novel hypotheses. And obviously, this study is far from testing or proving any of the existing hypotheses. They can just provide suggestive information.
Line 208. After using arrows and labels on Figure 2, this will be more clear. It seems that unlike in humans, the cartilage extends into the bony ET beyond istmus.

The cartilaginous tubular section extends into the bony isthmus. (eg. Graves 1944)

Line 256. “analyses”

Corrected

Line 451.Figue 1. Great demonstration of the methods.

Line 463.Figure 2. It is better to use labels in the bottom images, to identify the bone, cartilage, muscles.
Line 468-Figure 3. Additional combined images (top) from different perspectives (top down, bottom up, lateral to medial (perpendicular to this one) will enhance the reader’s perception.
Line 475. Figure 4. Additional fusion images that include the 2 key muscles will be useful.
Line 490-Fig 6. It would be great to have this figure in two other dimensions, perpendicular to the ET orifice axis, to better appreciate the relationship of the LVPM and the ET cartilage, to better emphasize the “functional” implications of this relationship. Need to be clear also is that this is not a functional model, this statis model though has functional implications due to the presumed changes in the vector and the length. This concept is hard for those readers not familiar with the functional anatomy, so need to expand on this in the text and on the figure.
It is also best to include this ET cartilage and LVPM relationship in multiple images starting from the nasopharyngeal orifice extending deeper, to demonstrate how this relative position shifts, for additional supportive information for the presumed “functional” implications.
Such images will be much more useful than those in Figure 5.

The Figures have been extensively expanded following the Editor's valuable comments, and hopefully now suffice for illustration to a more suitable degree.

Dr. Robert Schuon
